# Clusterin as a New Marker of Kidney Injury in Children Undergoing Allogeneic Hematopoietic Stem Cell Transplantation—A Pilot Study †

**DOI:** 10.3390/jcm9082599

**Published:** 2020-08-11

**Authors:** Kinga Musiał, Monika Augustynowicz, Izabella Miśkiewicz-Migoń, Krzysztof Kałwak, Marek Ussowicz, Danuta Zwolińska

**Affiliations:** 1 Department of Pediatric Nephrology, Wrocław Medical University, 50-556 Wrocław, Poland; monika.augustynowicz@umed.wroc.pl (M.A.); danuta.zwolinska@umed.wroc.pl (D.Z.); 2 Department of Bone Marrow Transplantation, Oncology and Pediatric Hematology, Wrocław Medical University, 50-556 Wrocław, Poland; imiskiewicz@usk.wroc.pl (I.M.-M.); krzysztof.kalwak@umed.wroc.pl (K.K.); marek.ussowicz@umed.wroc.pl (M.U.)

**Keywords:** acute kidney injury, cystatin C, hyperfiltration, kidney injury molecule (KIM)-1, tubular damage

## Abstract

Background and aims: The markers of renal damage defining subclinical AKI are not widely used in children undergoing allogeneic hematopoietic stem cell transplantation (alloHSCT). The aim of the study was to evaluate serum and urinary clusterin as indices of kidney injury after alloHSCT in relation to damage (kidney injury molecule (KIM)-1) and functional (cystatin C) markers. Material and methods: Serum and urinary clusterin, KIM-1 and cystatin C concentrations were assessed by ELISA in 27 children before alloHSCT, 24 h, 1, 2, 3 and 4 weeks after alloHSCT and in controls. Results: All parameters were significantly higher in HSCT patients compared to controls even before the transplantation. The serum concentrations increased after HSCT and this rising trend was kept until the third (clusterin) or 4th (KIM-1, cystatin C) week. Urinary clusterin and KIM-1 were elevated until the third week and then decreased yet remained higher than before HSCT. Urinary cystatin C has risen from the second week after HSCT and decreased after the third week but was still higher than before alloHSCT. Conclusions: The features of kidney injury are present even before alloHSCT. Clusterin seems useful in the assessment of subclinical AKI and may become a new early marker of sublethal kidney injury in children.

## 1. Introduction

Renal tubular epithelial cells are prone to hypoxia and metabolic stress, thus they become first target cells in the course of kidney injury. Contrast-induced nephropathy is a classic example of reversible acute kidney injury (AKI) with tubular involvement [1]. Animal and human studies showed that contrast administration triggers both systemic and renal cytotoxic effects [2,3]. However, if the conditions are unfavorable, further irreversible changes may lead to progression to chronic kidney disease [4].

Acute kidney injury is a well-documented phenomenon characteristic for HSCT [5,6,7,8]. However, most studies on AKI take into account the KDIGO classifications, focusing on the serum creatinine values and diuresis [9]. Such criteria do not ease the AKI diagnosis. In order to secure the patient with positive fluid balance and prevent oliguria, additional hydration and forced diuresis are implemented. These conditions may bias the values of estimated glomerular filtration rate (eGFR) and urine output. Recent classifications have expanded the definition of AKI beyond the functional criteria. They distinguish four options, based on the combined evaluation of function and damage markers [10]. This new approach defines normal renal function as an absence of any index alteration, subclinical AKI as an isolated increase of any damage marker, functional AKI when solely function markers are modified and combined AKI with both function and damage markers altered [10,11].

The search for new markers was conditioned by the failure of serum creatinine as an early marker of renal function decrement. The indices of cellular damage—especially of tubular injury—are of particular interest as new markers of the so-called “subclinical AKI” [10]. The preliminary studies concerning children after cardiosurgery or hematopoietic stem cell transplantation (HSCT) proved that combination of the biomarkers of renal function and tubular damage may be of added value in the early diagnosis of AKI [12,13].

Indeed, the risk of AKI is increased in children undergoing hematopoietic stem cell transplantation (HSCT), mainly due to the nephrotoxicity of drugs. Additionally, renal hypoperfusion, infections and immune complications (including graft versus host disease) count. Moreover, patients with allogeneic HSCT suffer from AKI more often than those undergoing the autologous transplantation [14,15]. The assessment of renal function in the early (up to 28 days) post-transplantation period seems of paramount importance, because it may reveal the potential direction of future changes into either full renal recovery or acute kidney disease or chronic kidney disease [16,17]. First promising results in the population of children undergoing HSCT should urge further search for reliable early markers of kidney injury [13].

## 2. Aim of Study

Therefore, the objective of the study was to assess the usefulness of serum and urinary clusterin as new indices of kidney injury in the early post-HSCT period in relation to other renal damage (KIM-1) and functional (cystatin C) markers and to estimate their potential value as factors differentiating between children transplanted because of oncological and non-oncological reasons.

## 3. Material and Methods

### 3.1. Study Design and Settings

**Figure 1:**
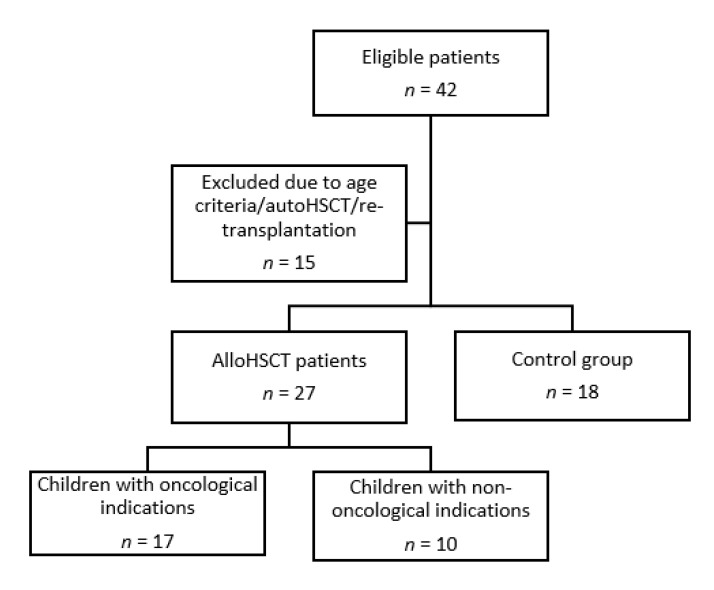
Patient flow.

This observational pilot study concerned 27 children (15 girls, 12 boys) undergoing first alloHSCT in the Department of Bone Marrow Transplantation, Pediatric Oncology and Hematology, in 2019 (patient flow is shown in Figure 1). The observation period started before introducing conditioning therapy, then parameter examinations were performed 24 h, 1, 2, 3 and 4 weeks after HSCT.

The exclusion criteria for the patients were the age below 2 years and over 18 years, autologous HSCT and retransplantation. The whole alloHSCT group contained 27 patients (median age 4.5 years, interquartile range 3.1–8.0 years). The subdivision into two groups was carried out depending on the indications for allotransplantation. Seventeen patients (median age 6.6 years, interquartile range 4.0–9.8 years) were qualified for transplantation due to oncological reasons, 10 (median age 4.5 years, interquartile range 3.1–7.0 years) underwent HSCT due to non-oncological indications (mainly severe aplastic anemia). In 79% of cases the donor was unrelated, in 18%-related and in 3%-haploidentical.

The conditioning therapy concerned myeloablative (busulfan, cyclophosphamide and fludarabine or fludarabine, treosulfan, thiotepa) or non-myeloablative (cyclophosphamide, fludarabine) regimens. In most patients graft versus host disease (GvHD) protocol contained pre-HSCT ATG, cyclosporine A since 1 day before HSCT and methotrexate given in the 1st, 3rd and 6th day after transplantation. Nineteen out of 27 patients developed GvHD. None of the patients died in the observation period.

The control group contained 18 age-matched children (9 girls, 9 boys; median age 7.8 years, interquartile range 7.0–9.8 years) with monosymptomatic nocturnal enuresis and normal kidney function.

Informed consent was obtained from the patients over 16 and their parents, if necessary.

### 3.2. Methods

Blood samples were drawn from peripheral veins after an overnight fast. Samples were clotted for 30 min, centrifuged at room temperature, 1000 g for 15 min, then serum was stored at −80 °C until assayed. Urine was collected aseptically from the first morning sample, centrifuged at room temperature, 1000 g for 15 min and then stored at −80 °C until assayed.

The serum and urine concentrations of clusterin, cystatin C and KIM-1 were evaluated by commercially available ELISA kits (clusterin EIAab, reagent kit E1180h; cystatin C R & D Systems, reagent kit DSCTC0; KIM-1 EIAab, reagent kit E0785 h). Standards, serum and urine samples were transferred to 96-well microplates precoated with recombinant antibodies to human clusterin, cystatin C, KIM-1 and creatinine. Captured proteins were then detected using monoclonal antibodies against clusterin, cystatin C and KIM-1 conjugated to horseradish peroxidase. Next, the assay was developed with tetramethylbenzidine substrate and blue color was developed proportionately to the amount of captured protein. The addition of acid stop solution ended the color development and converted it to the endpoint yellow. The intensity of the latter was measured in a microplate reader at 450 nm, with the correction wavelength at 550/650 nm. Each sample was tested in duplicate and the arithmetical mean was considered a final result. Measurements were performed according to the manufacturer’s instructions; results were calculated by reference to standard curves. Detection limits were as follows: clusterin 1.56 ng/mL; cystatin C 3.13 ng/mL; KIM-1 0.15 ng/mL. The intra-assay and inter-assay coefficients of variation (% CV) for examined parameters did not exceed 8.5% and 9.4%, respectively.

The assessment of kidney function relied on hematological protocols assessing serum creatinine in fixed time points. Serum and urine chemistry parameters were measured using automated routine diagnostic tests on the Beckman Coulter AU2700 analyzer. The serum creatinine was assessed with the use of enzymatic method (creatinine OSR61204 reagent, creatininase–sarcosine oxidase reactions). Serum and urine concentrations of all parameters was measured before conditioning, 24 h after allotransplantation and then 1 week, 2, 3, 4 weeks after alloHSCT. eGFR was calculated in all time points, based on the Schwarz formula [18]. The eGFR changes were confronted with the pre-transplantation values.

All urinary concentrations of evaluated parameters were normalized to urinary creatinine values.

AKI was diagnosed based on the pRIFLE criteria [9]. Hyperfiltration was defined as eGFR ≥ 140 mL/min/1.73 m^2^, according to recent meta-analysis and pediatric experience [19,20].

### 3.3. Statistical Analysis

Results were expressed as median values and interquartile ranges. The null hypothesis of normality of distribution of analyzed variables was rejected by Shapiro–Wilk test. Thus, the comparisons between paired and unpaired data were evaluated by using nonparametric tests (Friedman, Wilcoxon, Kruskal–Wallis, Mann–Whitney U). The correlations between parameters were assessed with the use of Spearman’s correlation coefficient R. Statistical analysis was performed using the package Statistica ver. 13.3 (StatSoft). A *p*-value < 0.05 was considered significant.

## 4. Results

None of the patients presented with eGFR < 60 mL/min/1.73 m^2^ and median eGFR values in both groups were above 90 mL/min/1.73 m^2^ at any time point (Table 1) Most oncological patients demonstrated hyperfiltration until the 3rd week after transplantation (with peak incidence in the first week after HSCT). Non-oncological children with eGFR > 140 mL/min/1.73 m^2^ were in minority. The median eGFR values in non-oncological children were comparable to those of the controls during the whole study except for the early (24 h after HSCT) measurement, when they became significantly higher (Table 1). Contrarily, the eGFR records in oncological patients remained significantly elevated compared to controls from point zero until the 3rd week after alloHSCT. They were increased throughout the whole study period compared to the non-oncological patients (Table 1).

**Figure 2:**
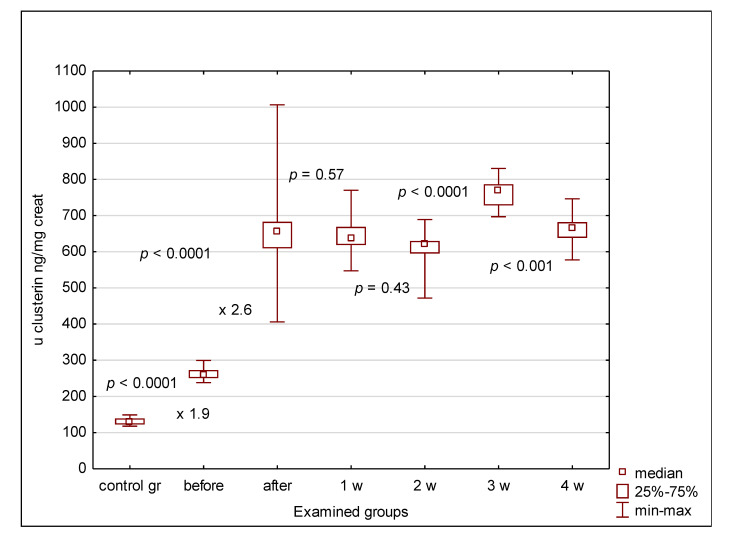
Urinary clusterin values in examined groups. before—before alloHSCT; after—24 h after alloHSCT; 1 w (2 w, 3 w, 4 w)—1 week (2, 3, 4 weeks) after alloHSCT.

**Figure 3:**
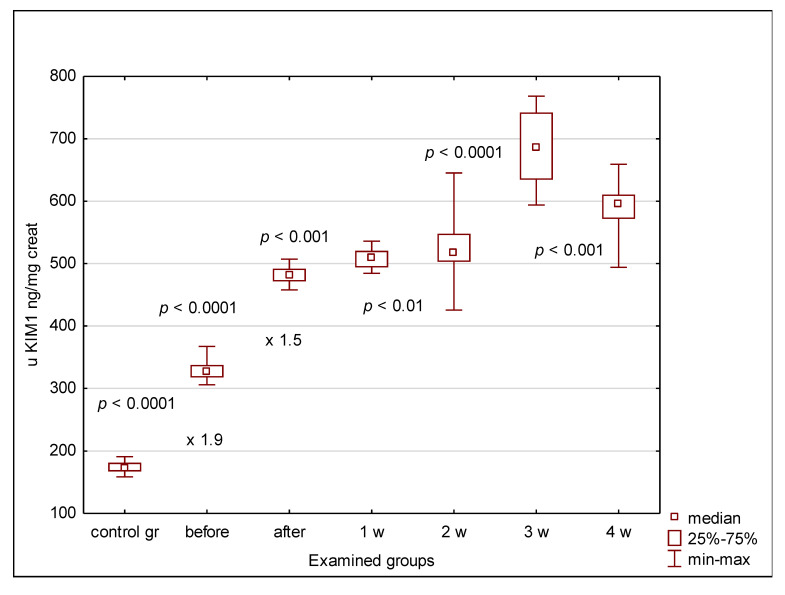
Urinary KIM-1 values in examined groups. before—before alloHSCT; after—24 h after alloHSCT; 1 w (2 w, 3 w, 4 w)—1 week (2, 3, 4 weeks) after alloHSCT.

**Figure 4:**
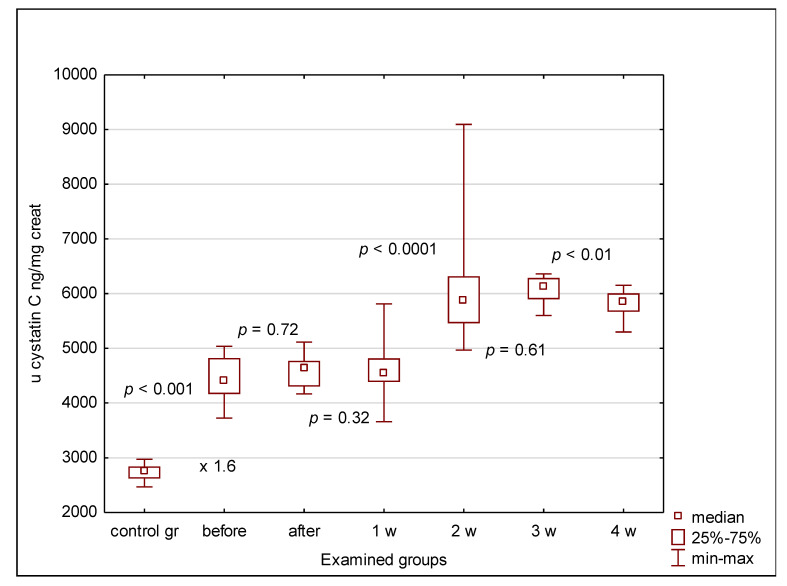
Urinary cystatin C values in examined groups. before—before alloHSCT; after—24 h after alloHSCT; 1 w (2 w, 3 w, 4 w)—1 week (2, 3, 4 weeks) after alloHSCT.

The urinary clusterin, KIM-1 and cystatin C concentrations were significantly elevated in all patients compared to controls, irrespective of the indication for transplantation (oncological or non-oncological), even before alloHSCT. Normalization of the urinary concentrations of clusterin, KIM-1 and cystatin C for urinary creatinine maintained these differences (Figure 2, Figure 3 and Figure 4). In the case of clusterin, the urinary values have increased nearly 3-fold 24 h after transplantation, then kept the plateau phase until the second week and rose again in the 3rd week. Finally, they decreased in the 4th week after HSCT, yet remained higher than before HSCT (Figure 2). The urinary KIM-1 values rose by 50% 24 h after HSCT, then kept growing until the 3rd week and finally decreased (Figure 3). Urinary cystatin C demonstrated the delayed elevation from the 2nd week after transplantation, lasting only until the 3rd week and followed by a significant decrease 1 week later (Figure 4). After 4 weeks of observation, all urinary biomarkers were still significantly elevated compared to the pre-transplantation values.

The serum concentrations of clusterin, KIM-1 and cystatin C in children before transplantation were significantly higher than in controls (Table 2). The serum values further increased 24 h after HSCT and the rise was most spectacular in the case of clusterin (over 2-fold) compared to 50%–60% elevation of other markers. Then the concentrations rose systematically until the 3rd week (clusterin) or 4th week (KIM-1, cystatin C) after alloHSCT. There was no significant difference in the urinary or serum marker values between oncological and non-oncological patients at any time point (Table 2).

AKI was diagnosed in 4 patients (3 oncological and 1 non-oncological), according to the pRIFLE criteria. Risk stage was diagnosed in 3 of them, 1 developed Injury stage. None of the patients required renal replacement therapy.

## 5. Discussion

Although AKI is a common finding in the patients undergoing alloHSCT, most of the classifications assessing the degree of renal impairment concentrate on the functional indices. However, damage markers seem a promising and objective alternative in the assessment of kidney injury. The fact that the number of potential AKI markers is increasing proves that the problem is emerging. Thus, our aim was to confront the known indices of tubular injury (KIM-1) and glomerular function (cystatin C, eGFR) with a new marker of cellular stress and damage. Clusterin has not been analyzed in a specific population of patients undergoing HSCT so far.

Every patient in this study group, irrespective of the indication for HSCT, demonstrated the features of cellular damage seen even before the procedure of transplantation. The interpretation of this unexpected result was quite challenging, because up-to-date studies on the populations undergoing HSCT have never compared their records to those of age-matched controls with normal renal function and no history of kidney injury. The pre-transplantation therapies, including chemotherapeutics and nephrotoxic drugs, seem of paramount importance as an explanation for this phenomenon. Indeed, all patients transplanted due to oncological reasons have undergone series of chemotherapies, whereas many of the non-oncological children were treated with potentially nephrotoxic antibiotics prior to alloHSCT. All these interventions were related to their primary diagnoses and could trigger the pre-transplantation subclinical kidney damage. Irrespective of the underlying cause, such observation would suggest that subclinical background is a common finding and, thus, the key player in the HSCT-related AKI.

The post-transplantation aggravation of cellular damage was rather predictable, taking into account the nephrotoxic and cytotoxic potentials of conditioning regimens, anti-GvHD prophylaxis and treatment of infections. The essential finding was that these signs of injury concerned both serum and urine, depicting not only renal, but also systemic cell damage after HSCT.

Among all analyzed parameters, the serum and urinary clusterin values have shown the most spectacular changes in children before HSCT compared to controls and before HSCT compared to 24 h after HSCT. The serum and urinary values before transplantation were at least two times higher than in controls and rose over 2.5-fold after transplantation. In the meantime, the other serum markers increased by no more than 60%, whereas the urinary values became 14% higher (KIM-1) or remained unchanged (cystatin C). Only in the case of clusterin the post-HSCT response was more spectacular than the difference between control group and pre-HSCT values.

Clusterin is a 75–80 kDa heat shock protein secreted by both epithelial and secretory cells in response to stress. Its protective and anti-apoptotic roles against renal ischemia-reperfusion injury have been demonstrated in murine kidneys [21]. Interestingly, clusterin was detected in both renal tubular epithelial and mesangial cells. Its decreased expression aggravated postischemic renal injury, as well as proteinuria in the course of glomerulopathy. Clusterin knockout mice suffered from the progression of renal inflammation and fibrosis after ischemia–reperfusion injury [22]. Investigation concerning humans is restricted to single reports on the urinary clusterin in patients with diabetes mellitus and promising results of a diagnostic multi-biomarker kit including urine clusterin in the scope of indices of chronic kidney injury [23,24].

Taking into account the experimental data, the above mentioned reports and our results, we could put forward the hypothesis about the protective role of clusterin in the kidney injury due to HSCT. Such presumption would provide the explanation for increasing levels in both serum and urine as a response to systemic and kidney stress conditions. Moreover, clusterin has turned out the most accurate marker predicting drug-induced AKI in adults, better than cystatin C or KIM-1 [25]. Interestingly, all three markers became higher compared to non-AKI controls already 1–3 days before the onset of AKI. This may suggest the reason for the early pre-transplant elevation of all indices compared to healthy controls. It also shows the added value of all tested markers in diagnosing nephrotoxicity. Moreover, yet, testing big cohorts may give unequivocal results in the pediatric population, questioning for example the accuracy of KIM-1 as a predictor of AKI in children [26].

In our study group, urinary KIM-1 behaved similarly to urinary clusterin, although the elevation after HSCT did not reach 50%. However, the rising trend persisted until the third week and then a significant decrease was noticed. Yet, the values remained higher than before transplantation. Such scheme would talk into the tubular damage aggravating since the HSCT procedure. The serum KIM-1 concentrations kept the systematic growth throughout the whole observation period, except for the plateau phase between 24 h and one week after HSCT. These results have suggested the ongoing tubular damage in the course of HSCT procedure, triggered most probably by nephrotoxic drugs. Increased serum KIM-1 could point at its release by the cells damaged in the course of conditioning therapy, anti-GvHD prophylaxis or current infection treatment, as well as the accumulation of a molecule that cannot be filtered freely through glomeruli due to its molecular mass of 90–110 kDa. The possible long-term consequence has been discovered in experimental studies, when chronic KIM-1 elevation in mice promoted fibrosis, thus linking AKI to CKD [27].

However, both abovementioned markers seemed similar in the mode of early reaction to kidney injury. The usefulness of clusterin and KIM-1 could be strengthened by the fact that their elevated serum and urine values were noticed even before transplantation, both in oncological and non-oncological patients, whereas pre-transplantation eGFR changes concerned only oncological children. In detail, their eGFR values were significantly higher compared to controls and most of the patients demonstrated hyperfiltration. This finding was confirmed by other authors, who put the main stress on hypermetabolic states and previous chemotherapy as causative factors of hyperfiltration [28]. The eGFR discrepancies could also be the consequence of many transplant-related covariates like catabolism, inflammation or weight loss, directly influencing serum creatinine [29].

Whether this hyperfiltration could be the early sign of progression into chronic kidney injury, remains unexplained, because the longer time of observation would be needed. However, the elevation of both damage parameters and eGFR could be the proof for the pre-transplantation kidney injury in oncological patients. This finding justifies the current attempts to prevent renal injury or at least to minimize the impact of potential nephrotoxins on the kidney. The reduction of nephrotoxic exposure is one of the effective tools already used in oncological patients [30]. The animal models suggest the possibility to prevent AKI with the use of phosphodiesterase-5 inhibitors prior to potentially nephrotoxic treatment [31,32]. Independent of the chosen strategy, the threat of renal injury should urge careful follow-up of the patient in order to avoid additional insults triggering irreversible damage to the kidney.

Cystatin Cis an established marker of glomerular function and a good predictor of AKI in children, so it seemed a good candidate to verify the abovementioned discrepant eGFR results. However, it did not confirm differences between oncological and non-oncological patients [33]. Out of the three examined markers, cystatin C was the only one with low molecular weight (13 kDa) freely filtered through glomeruli. Therefore, the fact that the elevation of cystatin C in serum has outrun the increase of the urinary value was the direct proof of the serum origin of cystatin C found in the urine. Having said that, the increased values of urinary clusterin and KIM-1, both of higher molecular weight than cystatin C, must have been of tubular cell origin. Therefore, their elevation was probably proportionate to the degree of cell damage and, in case of clusterin, to the intensity of protective mechanisms against kidney injury.

Summarizing, in our study clusterin has outperformed the targeted glomerular (cystatin C) and tubular (KIM-1) indices of kidney function. Therefore, it seems a promising early marker of the sublethal kidney injury, covering both tubular and glomerular spectrum of renal damage. Whether clusterin may become a duplex renal functional and damage marker, is yet to be established in the studies performed on a larger group of patients.

We also must acknowledge the limitations of our study. First, the clinical data were collected according to the hematological protocols, not taking into account all nephrological aspects. Thus, the full information about urine output is missing. This is the first report on the clusterin serum and urinary values in children, so it is impossible to confront them with the age-related reference values. We are also aware of the low number of patients and the heterogeneity of examined groups, which limits the power of conclusions and urges the continuation of the study throughout a longer period on a larger group of patients.

## 6. Conclusions

All children demonstrated the features of cell damage already before alloHSCT; thus, the subclinical AKI is a common finding in this population. Clusterin seems more useful in the assessment of subclinical AKI than the classical indices of tubular (KIM-1) and glomerular (cystatin C) damage analyzed separately. It may become a new early marker of sublethal kidney injury in this group of patients.

## Figures and Tables

**Table 1:** eGFR values in examined groups.

eGFR (mL/min/1.73 m^2^) Median Value (Interquartile Range)	Before alloHSCT	24 h after alloHSCT	1 Week after alloHSCT	2 Weeks after alloHSCT	3 Weeks after alloHSCT	4 Weeks after alloHSCT
Oncological patients	142 (112–149) ^a^	183 (153–216) ^a,b^	172 (155–205) ^a,b^	188 (166–195) ^a,b^	149 (140–174) ^a,b,c^	134 (123–149) ^a,c^
Non-oncological patients	107 (96–129)	140 (126–176) ^b^	131 (118–149) ^b^	130 (114–136) ^b^	129 (100–145) ^b^	126 (92–134)

^a^* p* < 0.05 oncological pts vs. non-oncological pts; ^b^ *p* < 0.05 any time point vs. before alloHSCT; ^c^ *p* < 0.05 2 weeks after vs. 3 weeks after; eGFR estimated glomerular filtration rate; alloHSCT allogeneic hematopoietic stem cell transplantation.

**Table 2:** Serum parameter values in examined groups.

Serum Parameter Median Value (Interquartile Range)	s Clusterin (ng/mL)	s KIM1 (ng/mL)	s Cystatin C (ng/mL)
Control group	1.3 (1.2–1.4)	2.3 (2.2–2.4)	148 (141.7–164.3)
Before alloHSCT	3.1 (3.0–3.1) ^a^	4.2 (4.1–4.3) ^a^	408 (387.6–433) ^a^
24 hafter alloHSCT	7.9 (7.2–8.4) ^a,b^	6.7 (6.3–6.8) ^a,b^	634.8 (604.6–689.1) ^a,b^
1 week after alloHSCT	8.9 (8.8–9.1) ^a,c^	6.7 (6.0–6.8) ^a^	905.6 (879–961.6) ^a,c^
2 weeks after alloHSCT	9.5 (9.3–10) ^a,d^	8 (7.8–8.2) ^a,d^	898 (878.7–926.3) ^a^
3 weeks after alloHSCT	13.3 (12.8–13.6) ^a,e^	8.9 (8.8–8.9) ^a,e^	1106 (1053–1157) ^a,e^
4 weeks after alloHSCT	13.3 (12.8–13.6) ^a^	9.2 (9.1–9.2) ^a,f^	1262 (1222–1288) ^a,f^

^a^*p* < 0.05 any time point vs. control group; ^b^ *p* < 0.05 24 h after alloHSCT vs. before alloHSCT; ^c^ *p* < 0.05 1 week after alloHSCT vs. 24 h after alloHSCT; ^d^ *p* < 0.05 2 weeks after alloHSCT vs. 1 week after alloHSCT; ^e^ *p* < 0.05 3 weeks after alloHSCT vs. 2 weeks after alloHSCT; ^f^ *p* < 0.05 4 weeks after alloHSCT vs. 3 weeks after alloHSCT. No significant correlations were detected between the analyzed parameters.
